# High Prevalence of *Borrelia miyamotoi* among Adult Blacklegged Ticks from White-Tailed Deer

**DOI:** 10.3201/eid2202.151218

**Published:** 2016-02

**Authors:** Seungeun Han, Graham J. Hickling, Jean I. Tsao

**Affiliations:** Michigan State University, East Lansing, Michigan, USA (S. Han, J.I. Tsao);; University of Tennessee, Knoxville, Tennessee, USA (G.J. Hickling)

**Keywords:** Borrelia miyamotoi, Ixodes scapularis, reservoir competence, tick-borne disease, white-tailed deer, zoonoses, vector-borne disease, Wisconsin

## Abstract

We compared the prevalence of *Borrelia miyamotoi* infection in questing and deer-associated adult *Ixodes scapularis* ticks in Wisconsin, USA. Prevalence among deer-associated ticks (4.5% overall, 7.1% in females) was significantly higher than among questing ticks (1.0% overall, 0.6% in females). Deer may be a sylvatic reservoir for this newly recognized zoonotic pathogen.

*Borrelia miyamotoi*, a relapsing fever group spirochete detected throughout the range of *Ixodes ricinus* complex ticks, has been implicated recently in human disease in the Northern Hemisphere (*1*–*3*). Enzootic maintenance of *B. miyamotoi*, however, has remained enigmatic since its discovery in Japan in 1995 (*4*). *B. miyamotoi* has been detected in several rodent species and their attached ticks, but the importance of rodents to the enzootic cycle remains uncertain (*5*,*6*). *B. miyamotoi* also has been detected in ticks feeding on European red deer (*Cervus elaphus*) (*7*); domestic ruminants (*8*); and recently by our group, in blacklegged ticks removed from white-tailed deer (*Odocoileus viginianus*) in the eastern United States (*9*). White-tailed deer are key hosts for adult *I. scapularis* ticks and a source of food for juvenile ticks. Thus, if deer are reservoirs for *B. miyamotoi*, in addition to maintaining tick populations, they may play a critical role in the epizootiology of this transovarially transmitted pathogen.

As an initial test of the hypothesis that white-tailed deer may be reservoir hosts, we collected questing and deer-associated adult *I. scapularis* ticks and assayed them for *B. miyamotoi*. We predicted that infection prevalence would be higher among deer-associated adult *I. scapularis* ticks than among questing adults. Furthermore, because females take larger blood meals than do males, we hypothesized that infection prevalence would be highest among deer-associated female ticks.

## The Study

All ticks were collected at Fort McCoy in central Wisconsin, USA, where *I. scapularis* ticks are well-established and where several *I. scapularis*–borne pathogens have been detected, including *Borrelia burgdorferi*, *Anaplasma phagocytophilum*, *Babesia microti*, and *B. miyamotoi* (*10*). Questing ticks were collected from vegetation at monthly intervals during April–November (2010–2012) by dragging or flagging as described in Rulison et al. (*11*). Adult ticks were collected from hunter-harvested deer in November 2010.

Total DNA was extracted from individual ticks by using the DNeasy Blood and Tissue kit (QIAGEN, Valencia, CA, USA) as described (*12*). We assayed for *B. miyamotoi* infection using a quantitative PCR that targets the 16S rDNA gene and can detect 1 spirochete per tick (*6**,**12*). To confirm the identity of positive samples, we sequenced a fragment of the 16S-23S intergenic spacer region (*12*). We then compared *B. miyamotoi* prevalence between groups, using a Fisher exact test; α = 0.05.

We tested 730 questing adult ticks and 355 adult ticks collected from 44 deer (33 males, 7 females, and 4 of undetermined sex) from 49 deer that were checked. Because prevalence of *B. miyamotoi* infection among questing adult ticks did not vary significantly by year (Table; p>0.05 for each sex), we pooled data across the years to increase statistical power.

**Table:** Prevalence of *Borrelia miyamotoi* among *Ixodes scapularis* ticks collected from white-tailed deer, Fort McCoy, Wisconsin, USA

Origin of ticks	Year	No. *B. miyamotoi*–positive ticks/no. tested (% positive)		
		M	F	Total
Questing on vegetation	2010	1/65 (1.5)	0/49 (0)	1/114 (0.9)
	2011	4/169 (2.4)	1/140 (0.7)	5/309 (1.6)
	2012	0/177 (0.0)	1/130 (0.8)	1/307 (0.3)
	2010–2012	5/411 (1.2)	2/319 (0.6)	7/730 (1.0)
Removed from deer	2010	5/199 (2.5)	11/156 (7.1)	16/355 (4.5)

*B. miyamotoi*–infected adult ticks were collected from 9 of the 44 tick-infested deer (20.5%, all males). Infestation with infected ticks was not significantly correlated with deer age or sex (both p>0.1). The infection prevalence among deer-associated adult *I. scapularis* ticks (4.5%), however, was significantly higher than that among questing adults (1.0%; p = 0.0004), and the infection prevalence among attached male ticks was significantly lower than that among attached female ticks (2.5% vs. 7.1%; p = 0.035, 1-tailed test). The infection prevalence among attached female ticks (7.1%) was 11.8× greater than that among questing female ticks (0.6%; p<0.0001, 1-tailed test).

We successfully sequenced a fragment of the intergenic spacer region from 34 of the 39 *B. miyamotoi*–positive samples. All sequences showed 99% similarity with published sequences for *B. miyamotoi* in GenBank (for example, accession no. AY363706). Four representative sequences have been deposited in GenBank (accession nos. KT321365–KT321368).

## Conclusions

Lyme borreliosis is the most common vectorborne disease in the United States; ≈30,000 new cases are reported to the Centers for Disease Control and Prevention each year (*13*). Given that *B. miyamotoi* uses the same vector ticks as *B. burgdorferi* and that the range of *I. scapularis* ticks continues to expand, it seems inevitable that the human population will be increasingly exposed to *B. miyamotoi* (*13*). *B. miyamotoi* has been found in rodents, but prevalence rates are so low that whether rodents play a key role as reservoirs is questionable (*6*).

If white-tailed deer are reservoir hosts for *B. miyamotoi*, adult ticks removed from deer would be expected to have a higher infection prevalence than sympatric host-seeking ticks collected from vegetation. Furthermore, because female *I. scapularis* ticks take larger blood meals than do males, the difference in prevalence among questing ticks should be most pronounced in female ticks. Both of these predictions are supported by our data: 1) the infection prevalence of adult ticks removed from deer was >4.5× that of questing adults, and 2) the infection prevalence of engorging females was >11× that of questing females.

Our data indicate that white-tailed deer at least permit *B. miyamotoi* to remain viable in the feeding ticks, in marked contrast to the situation with the Lyme disease agent, which is rapidly lysed by deer blood complement (*14*). Further research is needed to clarify how the ecoepidemiology of *B. miyamotoi* differs from that of *B. burgdorferi* and thus help inform public health management regarding diagnosis, treatment, and prevention of disease.

We note 2 caveats that arise from possible alternative explanations for the observed increase in *B. miyamotoi* infection prevalence among deer-associated adult ticks. First, spirochete numbers in infected questing adults could have been below the detection threshold of our quantitative PCR so that many questing ticks tested negative when they were positive (type II error). Then, as ticks were engorging, *B. miyamotoi* spirochetes may have reproduced sufficiently to rise above the threshold of detection. However, the mean PCR cycle threshold values at which *B. miyamotoi* was detected in questing ticks versus deer-fed ticks did not differ significantly (27.9 vs. 32.1, respectively; Wilcoxon rank sum test, p = 0.20). Thus we see no sign that false-negative rates would differ. Until more is known about the growth kinetics of *B. miyamotoi* in ticks that are engorging on a competent host, we cannot rule out this explanation. Nonetheless, if blood meal amplification occurs, it would strengthen the possibility that deer could be reservoir hosts.

The second caveat is that infection prevalence of ticks attached to deer could be elevated if venereal transmission of *B. miyamotoi* occurred during copulation. Preprandial mating has been documented in *I. scapularis* ticks, and mate guarding is common (*15*); however, that venereal transmission alone could produce the 4.5–11× increase in prevalence reported here seems unlikely.

If deer prove to be notable reservoirs for this pathogen, deer management practices to reduce tick populations and Lyme disease risk may provide additional health benefits by weakening *B. miyamotoi* transmission dynamics. Further supportive evidence for deer as a key reservoir could come in part from surveys of deer for infection with *B. miyamotoi* and by comparing infection prevalence of ticks removed from deer versus infection prevalence among ticks removed from other host species. A definitive answer, however, will require logistically challenging controlled transmission studies that quantify reservoir competence and characterize the course of infection in deer. The findings reported here point to the need for such studies.
